# Presence of a dog reduces subjective but not physiological stress responses to an analog trauma

**DOI:** 10.3389/fpsyg.2014.01010

**Published:** 2014-09-09

**Authors:** Johanna Lass-Hennemann, Peter Peyk, Markus Streb, Elena Holz, Tanja Michael

**Affiliations:** Division of Clinical Psychology and Psychotherapy, Department of Psychology, Saarland UniversitySaarbrucken Germany

**Keywords:** PTSD, animal assisted therapy, service dogs, stress, cortisol, trauma-film-paradigm

## Abstract

Dogs are known to have stress and anxiety reducing effects. Several studies have shown that dogs are able to calm people during cognitive and performance stressors. Recently, therapy dogs have been proposed as a treatment adjunct for post-traumatic stress disorder patients. In this study we aimed to investigate, whether dogs also have anxiety- and stress reducing effect during “traumatic stressors.” 80 healthy female participants were randomly assigned to one of four conditions. They were exposed to a “traumatic” film clip (trauma-film-paradigm). For one group of participants a friendly dog was present during the film, one group of participants was accompanied by a friendly human, another control group watched the film with a toy animal and the last group watched the film clip alone. Participants that were accompanied by the dog during the film reported lower anxiety ratings and less negative affect after the film clip as compared to the “toy dog group” and the “alone group.” Results of the “dog group” were comparable to the group that was accompanied by a friendly human. There were no differences in physiological stress responses between the four conditions. Our results show that dogs are able to lessen subjectively experienced stress and anxiety during a “traumatic” stress situation. This effect was comparable to that of social support by a friendly person. Implications for PTSD patients are discussed.

## INTRODUCTION

During the last decades animal assistance in (psycho)therapy and care has greatly increased. Recently, the employment of animal-assisted therapy and service animals (especially dogs) has been proposed as a treatment adjunct for post-traumatic stress disorder (PTSD) patients. PTSD is a severe psychological disorder that may develop after being confronted with a traumatic event and which is characterized by symptoms such as disturbing intrusive memories, avoidance of stimuli associated with the traumatic event, emotional numbing and hyperarousal (American Psychiatric Association [APA], 2013). Several programs in the USA established dog-assisted programs for PTSD patients and the anecdotal evidence of these programs is enthusiastic (Yount et al., 2012). PTSD service dogs seem to reduce stress and anxiety in stressful situations, especially when confronted with situations that remind the patients of the traumatic event (and thus might trigger intrusive reexperiencing).

Indeed, a large body of evidence has shown that dogs have anxiety and stress-reducing effects. These effects have been shown for physiological and endocrine parameters as well as subjective anxiety and stress ratings (for a review see Beetz et al., 2012b). Studies can be roughly divided into studies investigating stress parameters under resting conditions and studies investigating stress parameters in stressful situations. Studies investigating stress parameters under resting conditions showed that stroking and interacting with dogs reduced cortisol levels and physiological stress markers in healthy adults (Friedmann et al., 1983; Jenkins, 1986; Vormbrock and Grossberg, 1988; Odendaal, 2000; Odendaal and Meintjes, 2003; Barker et al., 2005; Handlin et al., 2011) and children (Friedmann et al., 1983) as well as physically impaired adults (Cole et al., 2007) and autistic children (Viau et al., 2010) as compared to control conditions. Studies investigating the influence of dogs on subjective and physiological stress parameters during stressful conditions have shown similar results: several studies in healthy adults have investigated whether the presence of a dog reduces stress responses during cognitive stressors: overall, these studies showed that dogs are able to reduce physiological and endocrine stress responses (Allen et al., 1991, 2002; Demello, 1999). Even though, in one study with healthy male students, dogs had no effect on heart rate and blood pressure during a stressful speech task (Straatman et al., 1997). In addition to the adult literature, several studies in children have shown that the presence of a dog reduced endocrine, physiological and subjective stress ratings. Stressors used in these studies were mild natural stressors such as physical examinations or dental examinations and social-cognitive laboratory stressors such as the TSST (Beetz et al., 2012a).

To summarize, programs implementing PTSD service dogs promote the stress and anxiety reducing effects of dogs, especially when patients are confronted with a reminder of the traumatic event. Studies in healthy adults and children as well as in some psychiatric disorders have shown that dogs have stress and anxiety reducing effects. However, these studies mainly employed cognitive and performance stressors and mild natural stressors. Thus, the aim of the present study was to investigate whether dogs also have stress and anxiety reducing effects during a “traumatic” stress situation. In our study, eighty healthy female participants were randomly assigned to one of four conditions. They were shown a “traumatic” film clip (trauma-film-paradigm). For one group of participants a friendly dog was present during the film, one group of participants was accompanied by a friendly person, another control group watched the film with a toy dog and the last group watched the film clip alone. The stress response to the traumatic film-clip was measured with subjective stress and anxiety ratings [State-Trait-Anxiety-Inventory-Trait (STAI-S) and Positive and Negative Affect Schedule (PANAS)] as well as physiological (ECG, Blood Pressure) and endocrine (saliva cortisol) measurements before, during and after the traumatic film. We expected participants in the “dog group” to experience less stress and anxiety in subjective as well as physiological and endocrine measurements as compared to the toy dog and the alone-group. Furthermore, we expected the anxiety reducing effects of the dogs to be comparable to that of social support by a friendly person, which has also been shown to reliably lessen stress responses (Cohen and Wills, 1985).

## MATERIALS AND METHODS

### PARTICIPANTS

Participants were 80 healthy female students at Saarland University, Germany, who responded to notices offering 20€ for taking part in a psychological experiment. Exclusion criteria were: fear of dogs, previous traumatic experiences, any axis I disorder or psychotherapeutic treatment, pregnancy and lactating. Due to assessment of cortisol, participation was restricted to healthy, non-smoking students with a body mass index of 20–25 kg/m^2^. In order to minimize the influence of menstrual cycle phase on hormonal status, only women with a regular use of monophasic oral contraceptives were included in the study. We also required participants to refrain from physical exercise, alcohol, and caffeinated drinks within 3 h prior to the experimental session. Experimental sessions took place between 2 p.m. and 6 p.m. to control for the diurnal cycle of cortisol. All participants gave their written informed consent. The research was approved by the ethical committee of the medical association of Saarland.

### MATERIALS AND MEASURES

#### Trauma film

The trauma film consists of an 11 min compilation of scenes from the film “Irreversible” directed by Gaspar Noe. It contains fictional scenes depicting physical and sexual violence. The trauma film has been used in previous studies (Nixon et al., 2009a,b) and has been shown to reliably induce physiological and subjective stress responses. Participants were informed in the study advertisement, letter of introduction, and informed consent process that the film clip contained graphic material which could be disturbing, and that they were free to withdraw at any time without penalty.

#### Subjective stress and anxiety measurements

***STAI-S***. We used the German version state scale of the STAI (Laux et al., 1981) to measure participants’ change in level of anxiety as a response to the traumatic film clip. The STAI is a brief self-report measure consisting of twenty items related to feelings of apprehension, nervousness, tension, and worry. Participants are asked to rate items on a scale from 1 to 4, where “1 = not at all” and “4 = totally agree.” The score for the STAI-S scale ranges from 20 to 80, while 20 indicates a very low state-anxiety level and 80 indicates a very high state-anxiety level. The internal consistency for the state scale of the STAI is high.

***PANAS***. We used the German version of the PANAS to assess changes in positive and negative mood before and after the trauma film (Krohne et al., 1996). The PANAS questionnaire consists of 10 positive affects (PA: interested, excited, strong, enthusiastic, proud, alert, inspired, determined, attentive, and active) and 10 negative affects (NA: distressed, upset, guilty, scared, hostile, irritable, ashamed, nervous, jittery, and afraid). Participants are asked to rate items on a scale from 1 to 5, on the basis of the strength of emotion where “1 = very slightly or not at all” and “5 = extremely.” For both of the PANAS domains, scores can range between 10 and 50. A higher score on the positive domain indicates greater PA. A higher score on the negative domain indicates greater NA. The validity and reliability of the PANAS questionnaire and its relationship between other measures of depression and anxiety was determined by Crawford and Henry (2004) showing that higher scores in NA is more related to anxiety, while lower scores in PA are more related to depression measures.

#### Physiological stress measurements

***Blood pressure***. Systolic and diastolic blood pressure was measured using a DINAMAP V100 device (GE-Healthcare, Munich, Germany) with a cuff placed around the upper arm. Blood pressure was measured three times (before film, during film (after 5.30 min had passed) and after the film clip).

***ECG***. To measure heart rate as response to the video a standard lead-II electrocardiogram (ECG) with two electrodes was used to collect a raw ECG signal with an ActiveTwo amplifier (BioSemi, Amsterdam, The Netherlands) at a sampling rate of 2048 Hz. R-waves were identified automatically by ANSLAB (Wilhelm and Peyk, 2012) and edited manually for artifacts, false positives or non-recognized R-waves and transformed into instantaneous heart rate.

***Cortisol***. Cortisol data was collected using Salivette tubes (Sarstedt). The participant first placed a cotton swab provided in each Salivette tube in her mouth and gently chewed on it for about 1 min. The swab was then placed back in the tube. Tubes were kept at -20°C until analysis. Saliva cortisol was analyzed at the cortisol laboratory of the University of Trier, Germany. After thawing the saliva samples for biochemical analysis, the fraction of free cortisol in saliva was determined using a time-resolved immunoassay with fluorometric detection, as described in detail elsewhere (Dressendorfer et al., 1992). For each participant the area under the curve with respect to increase (AucInc) was calculated (Pruessner et al., 2003). The AUCInc reflects the increase and decrease of cortisol levels over the entire sample time and takes into account individual differences in the initial cortisol levels of the participants.

#### Demographic data

***STAI-T***. Trait anxiety for all participants was assessed with the German version of the STAI-T (Laux et al., 1981). The STAI-T also contains 20 items measuring trait anxiety. Participants are asked to rate items on a scale from 1 to 4, where “1 = not at all” and “4 = totally agree.” The score for the STAI-T scale ranges from 20 to 80, while 20 indicates a very low trait-anxiety level and 80 indicates a very high trait-anxiety level. As described above reliability and validity have been shown to be high.

***Pet Attitude Scale***. The Pet Attitude Scale (PAS) is an 18-item scale measuring the general attitude toward pets (Morovati et al., 2008). Items are rated on a seven-point scale (from 1 = not at all, to 7 = totally agree). Participants can reach a maximal score of 126 and a minimal score of 18 points. The internal consistency and retest-reliability of the scale is high. Construct validity of the scale has also been shown to be high.

### EXPERIMENTAL CONDITIONS

#### Dog condition

Participants in the “dog condition” watched the trauma film accompanied by a trained therapy dog. Dogs were friendly looking trained therapy dogs of various breeds (flatcoated retriever, labrador retriever, carne corso, hovawart). All dogs were trained at the same therapy dog training center (Therapiehundezentrum Saar). Participants were briefly introduced to the dog before the experimental session started. They were allowed to feed the dog a treat and stroke the dog. The interaction with the dog was restricted to 2 min. During the film the dog was laying quietly beside the participant. The participant was allowed to establish physical contact with the dog during the trauma film. However, she was asked to constantly watch the video.

#### Toy dog condition

Participants in the “toy dog condition” watched the trauma film with a toy-dog. The toy dog was a life-size collie, which was seated next to the participants. Participants were briefly introduced to the toy dog before the experimental session started. The experimenter told the participants the name of the toy dog and spoke a little bit about the toy dog. This interaction was restricted to 2 min.

#### Friendly person condition

Participants in the “friendly person condition” watched the film clip together with a friendly female graduate student unknown to the participants. Participants were briefly introduced to the graduate student before the experimental session started. The graduate student introduced herself and talked with the participant for 2 min about neutral topics (e.g., weather, traffic).

#### Alone condition

Participants in the “alone condition” watched the film clip alone. The experimenter engaged them in a conversation about neutral topics for 2 min after they arrived at the laboratory.

### PROCEDURE AND DESIGN

The study took place at the laboratories of the Department of Clinical Psychology and Psychotherapy of the Saarland University. Participation included two appointments: an initial screening session clarifying study eligibility and the actual experimental session. Participants were assigned by an independent person according to a computer generated randomization list to one of the four conditions (dog, toy dog, friendly person, alone).

#### Screening interview

If participants fulfilled the inclusion criteria in a short telephone screening, they were invited to a first appointment in the laboratories: first, participants received an information sheet about the study, which informed them about the procedure and goals of the study. Afterward, participants gave their written informed consent. Then, inclusion and exclusion criteria where checked again and participants were asked to complete a demographic questionnaire, the STAI-T and the Pet Attitude Questionnaire. Afterward an appointment for the experimental session was made.

#### Experimental session

After arrival at the laboratories, participants were briefly introduced to the dog, the toy dog, the friendly person or the experimenter interacted shortly with the participant (as described in detail in: *experimental condition*). Afterward participants were led to the experimental chamber and electrodes for physiological measurements (ECG, BP) were attached (as described in detail in: *physiological stress measurement*). Participants were then asked to complete the PANAS and the STAI-S questionnaire (pre film measurement) and provided the first saliva sample. After completing the questionnaires, the dog, friendly person, or toy dog were seated beside the participant and the experimental procedure was started. Participants were told via computer screen that a 3-minutes-baseline measurement of physiological data would be collected and they should sit relaxed and look at the screen. After the baseline measurement participants were instructed that they would now be shown the video and that they should constantly watch the video and imagine they were an eyewitness of what was happening. Furthermore, the screen prompted them that they could withdraw from the experiment at any time if they decided to. Participants then started the video by pressing the “space” button. Physiological data was measured throughout the video. After the video had ended, participants were asked to provide another saliva sample and physiological measurement continued for three more minutes in which the participants were asked to sit relaxed and watch the screen (post measurement). After completing the post measurement, participants were asked to complete the PANAS and the STAI-S again. After this, the electrodes were removed and participants were led to another room, where they provided four more saliva samples (15, 30, 45, and 60 min after the video). After providing the last saliva sample, participants received 20 Euro for their participation and the experimenter briefly talked with the participant about the video. They were also given the possibility to pose further questions on the study design and goals of the study.

### STATISTICAL ANALYSIS

Data were analyzed by between-subject ANOVAs, with the alpha level set at *p* < 0.05. Effect sizes are reported as partial η^2^.

## RESULTS

### PARTICIPANT CHARACTERISTICS

The four groups consisted of 20 participants each. Three participants in the dog group had to be excluded from analysis (one due to data acquisition problems and two because of problems with the dogs (dog would not lie still beside the participant). Physiological data (ECG, Blood Pressure) was missing for two participants in the alone condition and one participant in the friendly person condition. Cortisol data was missing for one participant in the friendly person condition.

Groups did not differ in age [*F*(3,73) = 1.82, *p* = 0.15] or STAI-trait [*F*(3,73) = 1.71, *p* = 0.17] at pre-assessment. However, participants did differ in their attitude toward pets (PAS scores), with the toy dog group and the friendly person group showing a higher affinity for pets than the dog group and the alone group [*F*(3,73) = 11,94, *p* < 0.001]. Data are presented in **Table 1**.

**Table 1:** Participant characteristics.

	Dog condition (*N* = 17)	Toy dog condition (*N* = 20)	Friendly person condition (*N* = 20)	Alone condition (*N* = 20)	
	Mean (SD)	Mean (SD)	Mean (SD)	Mean (SD)	Significance
Age	24.65 (4.96)	21.60 (4.42)	22.60 (3.95)	22.35 (2.82)	*F*(3,73) = 1.82, *p* = 0.15
STAI-T	30.29 (5.45)	35.13 (7.95)	34.32 (8.01)	33.23 (5.37)	*F*(3,73) = 1.71, *p* = 0.17
PAS	72.99 (11.82)	89.08 (21.47)	95.16 (14.85)	69.71 (10.78)	*F*(3,73) = 11.94, *p* < 0.001

*STAI-T, State-Trait-Anxiety-Inventory-Trait; PAS, Pet Attitude Scale.*

### SUBJECTIVE STRESS RATINGS

To analyze the effects of experimental condition on subjective stress ratings, we conducted a 4^∗^2 MANOVA with experimental condition (dog, toy dog, friendly person, alone) as the between subject factor and time of measurement (pre-film measurement, post-film measurement) as within-subject factor. STAI-S, NA, and PA were dependent variables.

#### STAI-S

**FIGURE 1:**
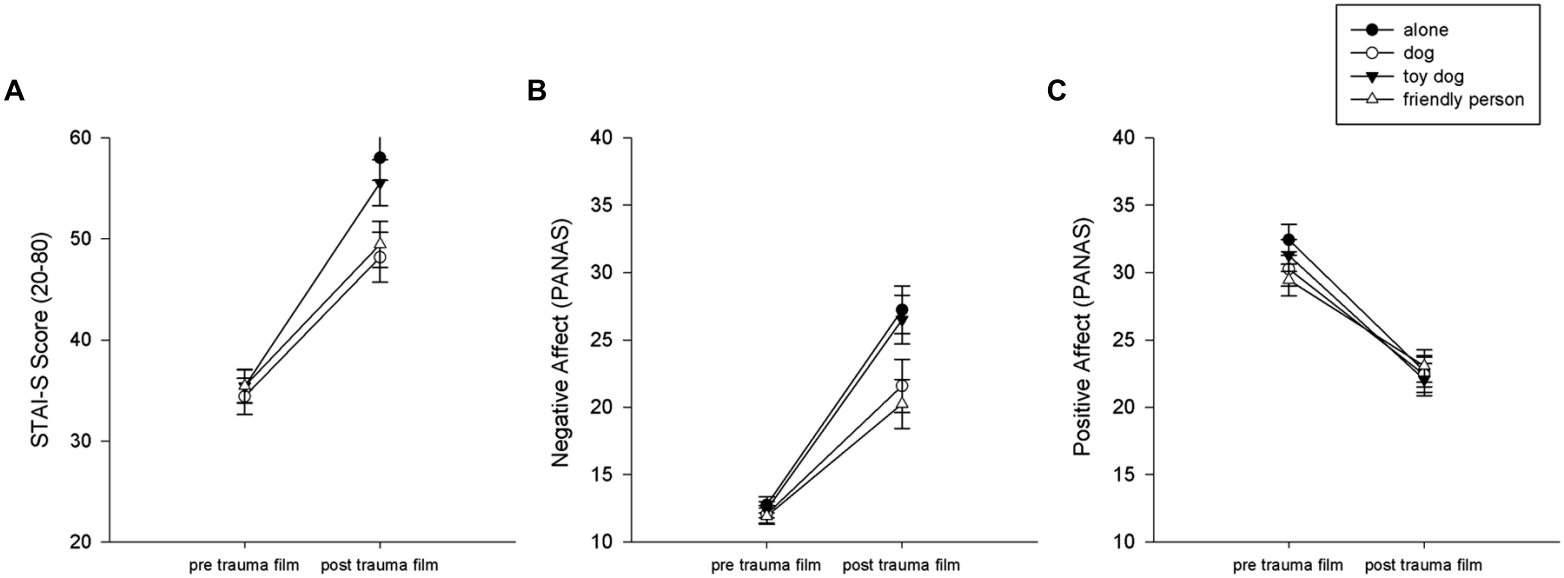
**Subjective stress and anxiety ratings (A: STAI-S, B: NA, C: PA) before and after the traumatic film clip in the different experimental conditions.** Error bars indicate one standard error of mean.

There was a main effect of time of measurement showing that anxiety levels rose for all four conditions from pre-film to post-film assessment [*F*(1,73) = 206.60, *p* < 0.001, η^2^ = 0.74]. However, there was a significant interaction between condition and time of measurement [*F*(3,73) = 3.16, *p* < 0.05, η^2^ = 0.115]. Participants in the dog group showed lower anxiety levels after the film as compared to participants in the alone group (*p* < 0.01) and the toy dog group (*p* < 0.05). Participants in the dog group and the friendly person group did not differ significantly from each other (*p* = 1.0). Data are presented in **Figure 1A**.

#### NA (PANAS)

For the negative affect scale of the PANAS there was also a main effect of time of measurement showing that negative affect rose from pre to post film assessment [*F*(1,73) = 169.56, *p* < 0.001, η^2^ = 0.70]. For the NA scale we observed the same interaction effect as for the STAI-S [*F*(3,73) = 2.96, *p* < 0.05, η^2^ = 0.11]: participants in the dog group showed lower anxiety levels after the film as compared to participant in the toy dog (*p* < 0.05) and alone group (*p* < 0.05). The dog group and the friendly person group did not differ significantly from each other. Data are presented in **Figure 1B**.

#### PA (PANAS)

For the positive affect scale of the PANAS there was also a main effect of time of measurement showing that positive affect declined from pre to post film assessment [*F*(1,73) = 138.18, *p* < 0.001, η^2^ = 0.65]. However, there was no significant interaction between condition and time of measurement for the positive affect scale [*F*(3,73) = 1.15, *p* = 0.33, n.s.]. Data are presented in **Figure 1C**.

### PHYSIOLOGICAL STRESS MEASUREMENTS

#### Cortisol

**FIGURE 2:**
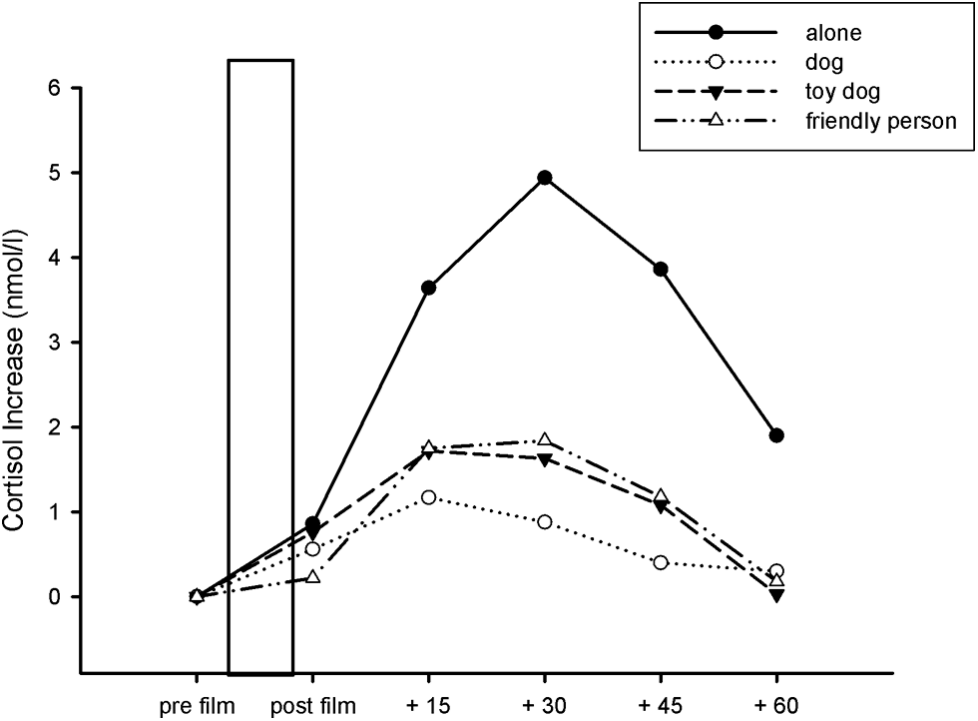
**Salivary cortisol in nanomoles per liter at several time points across the experiment in the different experimental conditions.** The bar represents the time of the trauma film. Error bars indicate one SE.

To analyze the effects of experimental condition on cortisol response to the trauma film we conducted a 4 (experimental condition: dog, toy dog, friendly person, alone)^∗^6 (time of cortisol measurement: pre film, post film, +15, +30, +45, +60) ANOVA. We found a main effect of time of cortisol measurement indicating a strong cortisol increase as a response to the trauma film in all four conditions [*F*(5,360) = 4.76, *p* < 0.001, η^2^ = 0.062]. However, there was no significant interaction between the four conditions and time of cortisol measurement [*F*(15,360) = 0.81, *p* = 0.49]. Data are presented in **Figure 2**. Because of baseline differences (pre film cortisol assessment) between the groups, we also analyzed the data with a one way ANOVA with experimental condition as the between subject factor and the AUCinc (which takes into account individual differences at baseline measurement) as dependent variable. The dog group had the lowest AUCinc [*M*(SD) = 2.87 (9.91)] followed by the friendly person group [*M*(SD) = 5.00(16.85)] and the toy dog group [*M*(SD) = 5.22 (10.44)]. Participants in the alone group showed the highest AUCinc [*M*(SD) = 13.35 (44.67)]. However, this descriptive difference was not significant [*F*(3,72) = 0.63, *p* = 0.59].

#### Blood pressure and heart rate

To analyze the effects of experimental condition on physiological responses to the trauma film, we conducted a 4 (experimental condition: dog, toy dog, friendly person, alone)^∗^3 (time of measurement: pre film, during film, post film) MANOVA with systolic blood pressure, diastolic blood pressure and heart rate as dependent variables. For systolic [*F*(2,140) = 24.94, *p* < 0.001, η^2^ = 0.27], diastolic blood pressure [*F*(2,140) = 8.99, *p* < 0.001, η^2^ = 0.11] and heart rate [*F*(2,140) = 24.33, *p* < 0.001, η^2^ = 0.26], there was a main effect of time of measurement showing an increase in blood pressure and heart rate during the film as compared to pre and post measurement. However, there was no significant interaction between experimental condition and time of measurement for systolic blood pressure [*F*(6,140) = 0.59, *p* = 0.73], diastolic blood pressure [*F*(6,140) = 0.87, *p* = 0.52] or heart rate [*F*(6,140) = 1.02, *p* = 0.41], indicating that experimental condition did not influence blood pressure or heart rate response to the trauma film. Data are presented in **Table 2**.

**Table 2:** Blood pressure and heart rate before, during and after the traumatic film clip in the four experimental conditions.

	Dog condition (*N* = 17)	Toy dog condition (*N* = 20)	Friendly person condition (*N* = 19)	Alone condition (*N* = 19)	
	Mean (SD)	Mean (SD)	Mean (SD)	Mean (SD)	Significance
**Systolic blood pressure**					*F*(6,140) = 0.59, *p* = 0.73
Pre film	125.00 (9.53)	123.55 (9.89)	125.37 (10.53)	120.89 (7.50)	
During film	127.82 (10.95)	128.55 (15.90)	125.37 (8.28)	123.94 (9.95)	
Post film	121.06 (8.57)	120.25 (11.02)	119.32 (10.26)	117.28 (9.60)	
**Diastolic blood pressure**					*F*(6,140) = 0.87, *p* = 0.52
Pre film	75.59 (7.87)	75.95 (8.26)	78.26 (9.30)	73.95 (4.18)	
During film	76.29 (9.08)	78.80 (7.97)	77.95 (4.09)	76.26 (6.38)	
Post film	73.94 (9.19)	76.20 (6.80)	74.37 (7.91)	71.42 (6.15)	
**Heart rate**					*F*(6,140) = 1.02, *p* = 0.41
Pre film	77.71 (11.41)	78.39 (12.05)	76.08 (9.65)	81.76 (11.20)	
During film	85.92 (20.23)	88.69 (16.63)	79.97 (8.71)	87.71 (14.80)	
Post film	80.80 (13.74)	79.70 (12.16)	74.40 (10.76)	82.11 (11.33)	

## DISCUSSION

The results of the present study indicate that dogs reduced subjectively experienced negative affect and anxiety after a traumatic film clip. Participants that were accompanied by a dog reported lower anxiety (STAI-S) and negative affect (PANAS, NA) scores than participants in the toy dog group and the alone group. Scores in the dog group were comparable to the scores of the social support group. To date studies investigating the stress and anxiety reducing effects of dogs primarily employed cognitive and performance stressors. Our data extend these findings to “traumatic” stress situations: dogs are also able to reduce stress and anxiety in “traumatic” stress situations.

For the positive affect scale of the PANAS, we did not find a significant interaction between experimental condition and time of measurement. All four groups showed lower PA after the traumatic film clip, but there was no significant difference between the four groups after the film clip. This might be due to the finding that PA is more related to depressive symptoms than to anxiety symptoms (Watson and Clark, 1988; Crawford and Henry, 2004) and our study protocol focused on inducing stress and anxiety. Furthermore, while the effects of a one session dog intervention on stress and anxiety are well documented, the effects of one session interventions on depressive symptoms are not as well investigated. To our knowledge there is only one study that investigated the effects of a one session animal assisted therapy on depressive symptoms before electroconvulsive therapy and this study showed that animal assisted therapy did have an inhibiting effect on anxiety symptoms but not on depressive symptoms (Barker et al., 2003).

Surprisingly, we did not find any significant difference between the four experimental conditions in physiological and endocrine stress markers. All groups showed a significant increase in physiological and endocrine parameters as a response to the traumatic film clip. However, experimental condition did not moderate the physiological and endocrine stress response. This stands in contrast to several studies showing reduced heart rate, blood pressure and a reduced cortisol increase as response to a stressor, when accompanied by a pet (Friedmann et al., 1983; Jenkins, 1986; Vormbrock and Grossberg, 1988; Odendaal, 2000; Odendaal and Meintjes, 2003; Barker et al., 2005; Handlin et al., 2011). However, some of these studies describe that the stress reducing effects of dogs are stronger when actual tactile contact (stroking the animal) is established and if the animal belongs to the participant (Odendaal, 2000; Odendaal and Meintjes, 2003). In our study, participants were allowed to stroke that dog, and nearly all participants actually established physical contact to the dog. However, in future studies one should have a closer look on the intensity of the contact, because this has been shown to be related to endocrine and physiological reactions. Another explanation for the diverging findings might be our stress induction method (trauma film), which differed from the stress tests used in previous studies.

One limitation of our study is that our sample consisted of women only. We decided to use women only, because of two reasons: first, in a (psychology-) student sample it is quite hard to realize sex-balanced samples and we therefore decided to investigate women only. Second, the traumatic film clip includes a rape scene which is thought to have a larger impact on women than on men. Because we were interested in the moderating effect of our experimental conditions on stress responses to the trauma film and not in sex differences in stress responses to the trauma film, we decided to investigate women only. However, future studies should extend these findings to both genders.

A further limitation of our study is that we cannot directly transfer our findings to PTSD patients. Even though many people experience a traumatic event during their life time, only a small proportion of those develops PTSD as a response to the traumatic event. However, using the trauma film paradigm, we are only able to mimic stress and anxiety responses to a “traumatic” situation and no real life PTSD symptoms. Nevertheless our study is the first to show that dogs have anxiety reducing effects during such a situation.

Furthermore, it has to be mentioned that our groups did have baseline differences in their attitudes toward pets as measured with the PAS (Morovati et al., 2008). Attitude toward pets is thought to be one factor that moderates the effects of dogs on psychological and physiological parameters. Importantly, the attitude toward pets was highest in the toy dog group and the friendly person group as compared to the dog group and the alone group. Thus, the stress reducing effects in the dog group cannot be attributed to a moderating effect of a more positive attitude toward pets.

In future studies, the effects of dogs on intrusions after a “traumatic” film clip should be assessed. The trauma-film-paradigm is known to reliably induce intrusive memories in healthy participants (Holmes and Bourne, 2008). Due to the exploratory character of our study, we decided not to measure intrusive reexperiencing after the trauma film. However, it is very interesting and highly relevant to analyze, whether the presence of a dog during the trauma film also reduces intrusion frequency as a response to the traumatic film. It would also be interesting to investigate the effects of dogs on recovery after the traumatic film clip, e.g., presence a dog in the recovery phase after the traumatic film clip, because there is evidence that dogs lead to a faster recovery from stress and anxiety symptoms. This would also be a preliminary hint for the employment of dog assisted interventions as emergency aid directly after traumatic events.

Post-traumatic stress disorder is a severe psychological disorder. Service dogs have been proposed as a promising treatment adjunct for PTSD patients (Yount et al., 2012). In our study we showed that dogs reduced subjective stress and anxiety in a “traumatic” situation. The effects of dogs were comparable to those of social support by a friendly person. It is important to note that social support is an important factor in buffering PTSD-symptoms (Zoladz and Diamond, 2013). However, one symptom of PTSD is that patients feel detached from others and emotionally numb (American Psychiatric Association [APA], 2013). Thus, PTSD patients are often not able to get social support from other people. Anecdotal evidence suggests that PTSD patients often find it easier to establish good relationships with animals and as our data suggest these may have similar stress buffering effects in situations that remind the patients of the traumatic event. In summary, the current investigation demonstrates that dogs do not only lead to a stress reduction during cognitive and performance stressors, but also during “traumatic” stress situations, thereby providing preliminary support for the idea that service dogs may serve as a useful treatment adjunct in PTSD patients by reducing anxiety and stress, when patients are confronted with a reminder of the traumatic event.

## Conflict of Interest Statement

The authors declare that the research was conducted in the absence of any commercial or financial relationships that could be construed as a potential conflict of interest.
